# Supplementary material for: Detection of CTNNB1 Hotspot Mutations in Cell-Free DNA from the Urine of Hepatocellular Carcinoma Patients
Source: Diagnostics (Basel). 2021 Aug 14;11(8):1475. doi: 10.3390/diagnostics11081475 (PMC8393790; doi:10.3390/diagnostics11081475)
Supplement: Supplementary file 1 [file diagnostics-11-01475-s001.zip › diagnostics-1299517-supplementary.pdf]

Supplemental Table S1. Frequency of *CTNNB1* mutation in exon 3.

	CTNNB1 exon 3 codons (number of occurrences listed from each reference)																														
**Reference Data**	**23**	**31**	**32**	**33**	**34**	**35**	**36**	**37**	**38**	**39**	**40**	**41**	**42**	**43**	**44**	**45**	**46**	**52**	**55**	**69**	**136**	**185**	**333**	**335**	**338**	**368**	**383**	**387**	**405**	**757**	**Deletions**	**Insertions**
Cbioportal.org		1	22	19	6	0	5	11	0	0	0	12	0	0	0	10	1		1		1	1	1	8	1	1	2	4	1	1	4	1
Ding, X., et al. *PLoS One* . 2014			2		3			3				5				2															0	0
Taniguchi, K., et al. *Oncogene* . 2002			1	1	2							1				8															1	1
Chun M. Wong, C.M., et al. *Cancer* . 2001					1	1	1					1				2															1	0
Laurent-puig, P., et al. *Gastroenterology* . 2001	1		2	4	2	1	1	2				5				10															2	0
Li-Chun, L., et al. *Oncology* . 2014	1	1	3	2	3			1				2	1			1															0	0
Galy, O., et al. Hepatitis *Research and Treatment* . 2011				2				2				2																			0	0
Madeleine A., et al. *Molecular Cancer* . 2008				1		1	1	2				2			1	2															0	0
Huang, H., et al. *Am J Path* . 1999			3	1				3								2															0	0
Legoix, P., et al. *Oncogene* . 1999			3	3	1	1		1				4				8															2	0
Nhieu, JTV., et al. *Am J Pathol* . 1999			1	1	2		1				1	2				1															2	0
Terris B., et al. *Oncogene* . 1999			3	3	1			1								2															5	0
% Frequency of mutation out of compiled reference data	0.8	0.8	15.9	14.7	8.4	1.6	3.6	10.4	0.0	0.0	0.4	14.3	0.4	0.0	0.4	19.1	0.4	0.0	0.4	0.0	0.4	0.4	0.4	3.2	0.4	0.4	0.8	1.6	0.4	0.4		
Total number of mutations out of compiled reference data	2	2	40	37	21	4	9	26	0	0	1	36	1	0	1	48	1	0	1	0	1	1	1	8	1	1	2	4	1	1	17	2

**Summary of compiled reference data**	
Total hotspot mutations region 1 (32-37)	137
Total hotspot mutations region 2 (41-45)	86
Total non-hotspot mutations	28
**Total number of overall mutations**	**251**
% Hotspot region 1	54.6
% Hotspot region 2	34.3
% Hotspot region 1 and 2	88.8

*Entire data analysis was compiled from data in reports listed above.

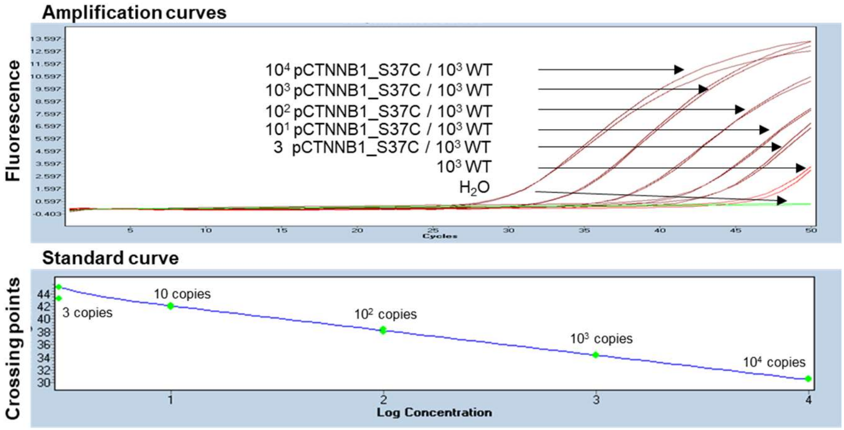

Supplemental Figure S1. Amplification curves of spiked-in standards and controls in the BNA^{NC}[NMe] clamp-mediated *CTNNB1* 32-37 mutation qPCR assay. The curves were generated with varying dilutions of the pCTNNB1_S37C plasmid as described in the Materials and Methods section. The analysis was carried out using the Roche LightCycler® 480 software.

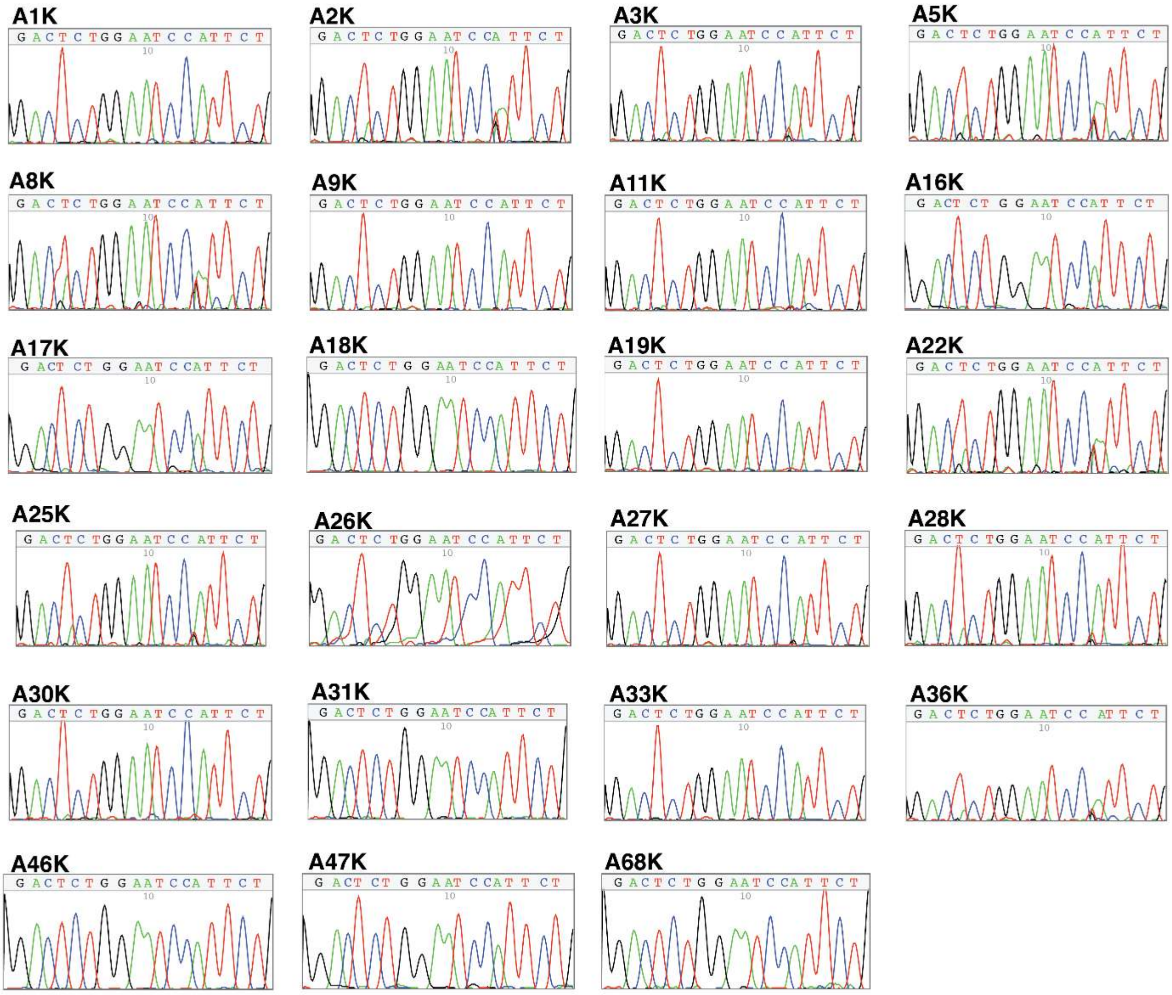

Supplemental Figure S2. DNA sequencing of HCC tissue samples negative for *CTNNB1* mutation. A subset of the samples that tested negative for *CTNNB1* mutations (23/55) were amplified with *CTNNB1*-hotspot-specific primers to generate a 213 bp product that was submitted for sequencing. The electropherograms depict the region of interest, codons 32-37 of exon 3 of the *CTNNB1* gene.
